# Proteomic profiling reveals mitochondrial dysfunction in the cerebellum of transgenic mice overexpressing DYRK1A, a Down syndrome candidate gene

**DOI:** 10.3389/fnmol.2022.1015220

**Published:** 2022-12-15

**Authors:** Mireia Ortega, Ilario De Toma, Álvaro Fernández-Blanco, Anna Calderón, Lucía Barahona, Ramón Trullàs, Eduard Sabidó, Mara Dierssen

**Affiliations:** ^1^Centre for Genomic Regulation (CRG), The Barcelona Institute of Science and Technology, Barcelona, Spain; ^2^Instituto de Investigaciones Biomédicas de Barcelona, IIBB/CSIC y Centro de Investigación Biomédica en Red, Barcelona, Spain; ^3^Department of Experimental Sciences, Universitat Pompeu Fabra (UPF), Barcelona, Spain; ^4^Centro de Investigación Biomédica en Red de Enfermedades Raras (CIBERER), Barcelona, Spain

**Keywords:** DYRK1A, cerebellum, mitochondria, oxidative phosphorylation system, Down syndrome, proteomics

## Abstract

**Introduction:**

DYRK1A is a dual-specificity kinase that is overexpressed in Down syndrome (DS) and plays a key role in neurogenesis, neuronal differentiation and function, cognitive phenotypes, and aging. Dyrk1A has also been implicated in cerebellar abnormalities observed in association with DS, and normalization of Dyrk1A dosage rescues granular and Purkinje cell densities in a trisomic DS mouse model. However, the underlying molecular mechanisms governing these processes are unknown.

**Methods:**

To shed light on the effects of Dyrk1A overexpression in the cerebellum, here we investigated the cerebellar proteome in transgenic Dyrk1A overexpressing mice in basal conditions and after treatment with green tea extract containing epigallocatechin-3-gallate (EGCG), a DYRK1A inhibitor.

**Results and Discussion:**

Our results showed that Dyrk1A overexpression alters oxidative phosphorylation and mitochondrial function in the cerebellum of transgenic mice. These alterations are significantly rescued upon EGCG-containing green tea extract treatment, suggesting that its effects in DS could depend in part on targeting mitochondria, as shown by the partially restoration by the treatment of the increased mtDNA copy number in TG non-treated mice.

## 1. Introduction

Trisomy of chromosome 21 causes Down syndrome (DS), the most common genetic cause of intellectual disability (Dierssen, 2012). DS individuals exhibit an overall reduction in brain size with a disproportionately greater reduction in cerebellar volume. Given that the cerebellum is a key regulator of motor control and motor learning, motor abnormalities in DS including the coordination of voluntary movement, gait, posture, and speech are ascribed cerebellar abnormalities. The analysis of single-gene contributions to DS has revealed the Dual-Specificity Tyrosine-Phosphorylation-Regulated Kinase 1A (*DYRK1A*), a DS candidate gene located in the 21q22.2 human chromosome region (Duchon and Herault, 2016), as a good candidate to contribute to cerebellar DS phenotypes. DYRK1A is expressed at high levels in the cerebellum (Martí et al., 2003), and mouse models with altered *Dyrk1A* expression exhibit altered motor abilities (Altafaj et al., 2001; Fotaki et al., 2002; Martínez de Lagrán et al., 2004). Overexpression of human *Dyrk1A* in post-mitotic cerebellar Purkinje neurons in zebrafish resulted in a structural disorganization of the Purkinje cells in cerebellar hemispheres and a compaction of this cell population that could be rescued by a novel selective DYRK1A inhibitor, KuFal194 (Buchberger et al., 2021). Similarly, normalization of the dosage of Dyrk1A in the partial trisomic Ts65Dn DS mouse model (Ts65Dn +/+/− with only two copies of Dyrk1A), rescues granular and Purkinje cell densities and the size of the granular and molecular layers (García-Cerro et al., 2018). Interestingly, *in vitro* studies have shown that bioenergetic dysfunction contributes to the reduced neurogenesis in progenitor cells from Ts65Dn, that is rescued by polyphenol 7,8-dihydroxyflavone (Valenti et al., 2021) suggesting a critical role of mitochondrial dysfunction in neurodevelopmental alterations in DS (Valenti et al., 2017). Indeed, we have previously shown that green tea extract containing epigallocatechin-3-gallate (EGCG), the main polyphenol in green tea, rescued the proteomic hippocampal alterations in both trisomic and *Dyrk1A* overexpressing transgenic (TG) mice (De Toma et al., 2019, 2020).

To elucidate putative mechanisms of the role of *Dyrk1A* in the cerebellum, here we explored the proteome changes induced by its overexpression in TG mice, and their possible restoration upon treatment with green tea extract containing EGCG, which has DYRK1A kinase inhibitor properties. We detected important proteomic alterations in TG cerebellum with over 200 proteins showing changes in abundance. The most significant changes indicate mitochondrial dysfunction as a crucial factor in the pathogenesis of cerebellar dysfunction in TG mice. Interestingly, treatment with green tea extracts rescued the level of many proteins, especially those involved in mitochondrial function. Thus, we also explored whether mitochondrial malfunction in a neuron-rich area of the cerebellum of TG mice could be recovered upon treatment with green tea extract containing EGCG. Our findings suggest that increased mtDNA copy numbers in TG non-treated mice are partially restored by EGCG-containing green tea extract.

## 2. Materials and methods

### 2.1. Animal models

All the experiments were performed using 2-month male wild-type (WT) and transgenic mice overexpressing Dyrk1A (TG). We selected this stage because most of the alterations found in TgDyrk1a mice such as memory and motor deficits (Altafaj et al., 2001) are already present at 2 months. Neuronal alterations such as reduced dendritic arborizations, reduction of neurite outgrowth, synaptogenesis, and spontaneous activity have also been described at this stage (Martínez de Lagran et al., 2012). Mice were obtained by crossing TG male mice with C57BL6/SJL WT female mice. Mice were reared in standard cages (20 × 12 × 12 cm Plexiglas cage) in groups of 2–3 animals and maintained under a 12-h light–dark cycle (8:00 h to 22:00 h) in controlled environmental conditions of humidity (60%) and temperature (22 ± 1°C) with *ad libitum* access to food and water. All procedures were approved by the local ethical committee (Comité Ético de Experimentación Animal del PRBB (CEEA-PRBB; MDS 0035P2), and met the guidelines of the local (law 32/2007) and European regulations (EU directive e no. 86/609, EU decree 2001-486) and the Standards for Use of Laboratory Animals no. A5388-01 (NIH). The CRG is authorized to work with genetically modified organisms (A/ES/05/I-13 and A/ES/05/14). Animal del PRBB (CEEA-PRBB); MDS 0035P2), and met the guidelines of the local (law 32/2007) and European regulations (EU directive e no. 86/609, EU decree 2001-486) and the Standards for Use of Laboratory Animals no. A5388-01 (NIH). The CRG is authorized to work with genetically modified organisms (A/ES/05/I-13 and A/ES/05/14).

### 2.2. EGCG-containing green tea extract treatment

One month-old TG or WT mice were assigned using a simple randomization to either control conditions or a treatment consisting on green tea extract containing 45% EGCG for 30 days. Green tea leaf extract was used to create a green tea extract with 45% EGCG, which was then used to make new EGCG solutions every 2 days to be added to drinking water (EGCG dosage: 0.326 mg/ml, 0.65 mg per day, and 30 mg/kg per day; Mega Green Tea Extract, Decaffeinated, Life Extension, Fort Lauderdale, FL; EGCG content 326.25 mg per capsule).

### 2.3. Mass-spectrometry-based proteomics

Mice were sacrificed, and the dissected cerebelli were frozen at −80°C. For the experiments, tissues were homogenized with a RIPA-modified buffer (50 mM tris-HCl pH 7.5, 150 mM NaCl, 1 mM EDTA, 1% NP-40, 0.1% sodium deoxycholate with the addition of 5 mM b-glycerophosphate, 10 mM sodium fluoride, 10 mM sodium orthovanadate, and protease inhibitors from the Complete Protease Inhibitor Cocktail Roche). Samples were sonicated using Bioruptor^®^ (Diagenode) for 5 min with 30 on/off cycles, maintaining the samples on ice, and were centrifuged for 10 min 10,000 rpm at 4°C. Proteins from the supernatants were precipitated overnight at −20°C by adding a volume of ice-cold acetone in six-fold excess. The acetone-precipitated proteins were solubilized in a denaturation buffer (6 M urea and 200 mM ammonium bicarbonate in water). Final protein content was quantified using the BCA assay (Pierce). Proteins were reduced with dithiothreitol (DTT, 10 mM, 37°C, 60 min), and alkylated with iodoacetamide (IAM, 20 mM, 25°C, 30 min). Then samples were diluted with 200 mM ammonium bicarbonate up to 2 M urea, digested overnight with Lys-C at 37°C, and then diluted two-fold again and digested overnight with trypsin at 37°C. Peptides were desalted using a C18 MicroSpin 300A silica column (The Nest Group, Inc.), evaporated to dryness using a SpeedVac, and dissolved in 30 μl of 0.1% formic acid in water.

### 2.4. Liquid chromatography–tandem mass spectrometry

For each sample, 1 μg of tryptic peptides from digested cerebellar tissue was injected in an LTQ-Orbitrap Velos Pro mass spectrometer (Thermo Fisher Scientific) coupled to a nano-LC (EASY-nLC, Proxeon). Nano-LC was equipped with a reversed-phase chromatography column of 25 cm with an inner diameter of 75 μm, packed with 3 μm C18 particles (Nikkyo Technos, NTCC-360/75-3-25L), and a Nano Trap Column Acclaim PepMap100 100 μm × 2 cm C18, 5 μm, 100A (Thermo, 164,199). Chromatographic gradients started at 93% of buffer A and 7% of buffer B with a flow rate of 250 nl/min during 5 min and linearly changed to 65% buffer A and 35% buffer B after 240 min. After each analysis, the column was washed for 16 min with 90% buffer A and 10% buffer B (Buffer A: 0.1% formic acid in water. Buffer B: 0.1% formic acid in acetonitrile). The mass spectrometer was operated in positive ionization mode with the nanospray voltage set at 2.2 kV and the source temperature at 250°C. Ultramark 1621 for the FT mass analyzer was used for external calibration prior to the analyses. The background polysiloxane ion signal at m/z 445.1200 was used as lock mass. The instrument was operated in data-dependent acquisition (DDA) mode with 1 microscan at resolution of 60,000 at 400 m/z and survey scans were recorded over a mass range of m/z 350–2,000 with detection in the Orbitrap mass analyzer. Auto gain control (AGC) was set to 10^6^, dynamic exclusion was set at 60 s, and the charge-state filter disqualifying singly charged peptides for fragmentation was activated. Following each survey scan, the top 20 most intense ions with multiple charged ions above a threshold ion count of 5,000 were selected for fragmentation at normalized collision energy of 35%. Fragment ion spectra produced *via* collision-induced dissociation (CID) were acquired in the linear ion trap, AGC was set to 5 × 10^4^, and isolation window of 2.0 m/z, activation time of 0.1 ms, and maximum injection time of 100 ms were used.

### 2.5. Mass spectrometry data analysis

Acquired mass spectra were processed using the MaxQuant computational platform version 1.5.2.8 (Cox and Mann, 2008). The MS2 spectra were searched by using the Andromeda search engine (Cox et al., 2011) against the UniProt sequence database for Mus musculus (17,263 forward entries; version from July 2015). The search included cysteine carbamidomethylation as a fixed modification, and N-terminal protein acetylation and methionine oxidation as variable modifications. We allowed a maximum of two miscleavages, 4.5 ppm as mass tolerance for precursor ions, and 0.5 Da as mass tolerance for fragment ions. FDR was set to 1% at the peptide and protein level, and protein identification required at least one unique or razor peptide per protein group. The MaxQuant algorithm was used to retrieve extracted ion currents (XICs) per peptide feature for quantification purposes. Areas under the curve for each peptide were calculated and later used to estimate protein intensities during the statistical analysis. Statistical analysis was performed using the R package MSstats version 2.6.0 (Choi et al., 2014). One of the five biological replicates of the TG.NT group was excluded from the analysis, because the number of peptides identified was substantially lower (e.g., 1,000 peptides) than the average of peptides identified in the whole experiment. To ensure high confidence in our quantitative data, only peptides observed at least in three of the five biological replicates (or at least in two when we remained only with four biological replicates), were used, and no imputation of missing values was performed.

#### 2.5.1. Differentially expressed proteins

Downstream bioinformatics analysis was performed on proteins that showed a significant change in abundance with a Benjamini adjusted *p*-value lower than 0.05 and a log2(Fold Change) (log2FC) >0.3 or lower than −0.3. The proteins preferentially detected present in one condition of the ones compared were added to the lists of differentially abundant proteins. A peptide was defined as “absent” when it was detected in <3 out of 5 biological replicates for a given condition (or <2 out of 4 biological replicates for the TG.NT group). Since blood contamination is a common problem in sample collection from dissected tissues, proteins belonging to the GO-term cell component “blood microparticle” were filtered out from all datasets before proceeding with downstream analyses.

We calculated the following contrasts for each of the three treatments were calculated (TG: TG mice; WT: wild-type mice; NT: not treated mice):

TG.NT-WT.NT (deregulated proteins in untreated TG mice)
TG.EGCG-TG.NT (proteins responding to the treatments in TG mice)
WT.EGCG-WT.NT (proteins responding to the treatments in WT mice)
(TG.EGCG-TG.NT) − (WT.EGCG-WT.NT) (interaction: proteins responding differently to the treatments in TG compared to WT mice)

For each protein whose abundance was significantly changing in the TG.NT-WT.NT contrasts we first calculated the genotype gap as the log2-fold-change obtained in the contrast TG.NT-WT.NT. Thereafter, we computed the “treatment gap” as the fold change observed in the TG.EGCG - WT.NT contrast. We therefore calculated the “percentage of recovery” based on the fraction of recovered abundance—in protein levels–after treatment—given by the difference between the genotype gap and the treatment gap—on the genotype gap: (genotype gap) – (treatment gap)/(genotype gap).

Note that once WT.NT is set to 0 (log2(1) = 0):

* TG.NT-WT.NT contrast can be simplified by TG.NT – log2(1) = TG.NT-0 = TG.NT.
* TG.EGCG - WT.NT = Levels impaired at the basal state + levels after treatment = (TG.NT-WT.NT) + (TG.EGCG-TG.NT) = TG.NT-0 + TG.EGCG –TG.NT = TG.EGCG.

And so:

(TG.NT − TG.EGCG)/TG.NT == [(TG.NT-WT.NT) − (TG.NT-WT.NT) + (TG.EGCG+TG.NT)]/(TG.NT-WT.NT)

The fraction of recovery could go from 0 (no recovery) to 1 (100% recovery). A value >1 indicates overcorrection. A value <0 is an impairment.

We considered as “rescued protein” those proteins with a % of recovery from 50% to 150%; as “overcorrected proteins,” the ones with a % of recovery higher than 150; “not sufficiently rescued” those proteins with a % of recovery between −50% and 50%; and “impaired proteins” those with a % of recovery < −50%.

With similar calculations, we computed the “percentage of impairment” by using instead of the TG.NT-WT.NT contrasts, its reverse WT.NT-TG.NT, in order to calculate how much the treatment in the wild type was reducing the differences between TG and WT.

#### 2.5.2. DYRK1A interactors

DYRK1A interactors were taken from the mammalian verified targets present in (Aranda et al., 2011; Duchon and Herault, 2016; Guard et al., 2019; Roewenstrunk et al., 2019). The significance of the overlaps between DYRK1A targets and the differentially abundant proteins was assessed using a Fisher Exact Test.

#### 2.5.3. Network analysis

The list of differentially abundant proteins was expanded to build a larger protein–protein interaction network including DYRK1A substrates and direct interactors. Specifically, for the disease network, we used the expanded list of proteins deregulated in the TG.NT-WT.NT contrast. For the expansion, we used a list of *bona fide* physical interactors mainly coming from the STRING (version 10; Szklarczyk et al., 2015) and an internal database from Interactome3d version 2017_01 (Mosca et al., 2013). We only considered interactions with a very high score (>0.9) in STRINGdb and IMEx index (Orchard, 2012; Orchard et al., 2012). Graph visualization was performed with the igraph R package using the Davidson Harel layout algorithm (Csardi and Nepusz, 2006).

#### 2.5.4. Enrichment analyses

Protein identifiers were annotated using the R packages UniProt.ws and biomaRt (Durinck et al., 2009). We assessed the significance of the overlaps using a Fisher’s exact test, *p*-values for the right tail are obtained directly using hypergeometric distribution considering as background the detected proteins for each contrast. For the Gene Ontology Enrichment analysis, we used the clusterProfiler R package (Yu et al., 2012).

#### 2.5.5. Identification of significant hubs

We used the poweRlaw R package (Alstott et al., 2014) to analyze the heavy tail distribution of interactions. We compared the power-law, Poisson, exponential, and log-normal distribution. Thereafter, we used the fitted distribution for calculating the *p*-values associated to each protein corresponding to encountering by chance a higher number of interactions per protein than the given protein, setting the threshold to define hub at a *p* < 0.05.

#### 2.5.6. Tissue-specific genes

Cerebellar-enriched proteins were detected using the TissueEnrich R package (Jain and Tuteja, 2019) processing RNA-Seq data across 17 mouse tissues (Mouse ENCODE Dataset; Shen et al., 2012).

We used TissueEnrich default parameters based on the algorithm from the HPA (Uhlén et al., 2015): genes with an expression level >1 TPM (Transcripts Per kilobase Million) or FPKM (Fragments Per Kilobase Million) that also have at least five-fold higher expression levels in a particular tissue (or group of tissues) compared to the average levels in all other tissues were defined as tissue enriched.

### 2.6. *cf*-mtDNA copy number and nuclear genomes using digital PCR

Quantification of mitochondrial DNA copy number per cell was performed with multiplex digital PCR as previously described (Podlesniy and Trullas, 2017). For the present studies we used two different primer pairs targeting opposite regions of the mtDNA molecule; one primer pair targeting the ND1 region (mtDNA-ND1: CTAGCAGAAACAAACCGGGC; CCGGCTGCGTATTCTACGTT), and another primer pair targeting the ND4 region (mtDNA-ND4: TAATCGCACATGGCCTCACA; GCTGTGGATCCGTTCGTAGT) of the mtDNA molecule. These primer pairs were selected with Primer-Blast for specific amplification of the mouse mtDNA reference sequence NC_005089.1.

The number of cells in the tissue homogenate was determined by quantification of diploid nuclear genomes with digital PCR by amplification of the single-copy nuclear gene BAX (forward, CACTGCCTTGGACTGTGTCT; reverse, CCTTTCCCCTTCCCCCATTC).

Results were expressed as mean ± standard deviation (SD). GraphPad Prism software v9 was used for statistical tests. Statistical analyses were performed using one-way analysis of variance (ANOVA) with Fisher’s least significant difference (LSD) *post-hoc* tests. Differences were considered statistically significant at a value of *p* < 0.05.

## 3. Results

### 3.1. Dyrk1a overexpression leads to protein dysregulation in the cerebellum disrupting mitochondrial function

**Figure 1:**
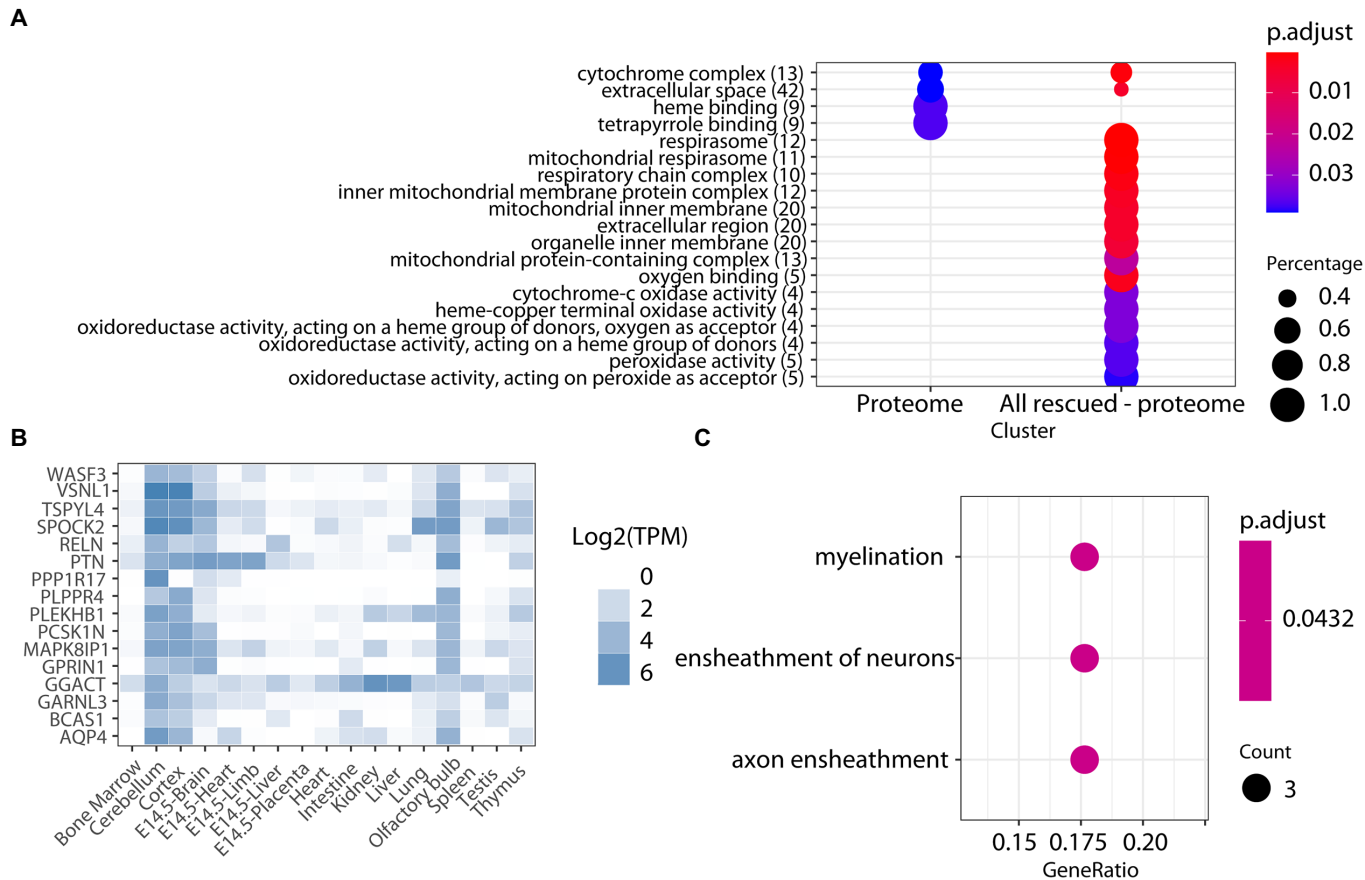
**(A)** Enrichment analysis for biological processes, molecular function, and cell components of proteins altered in the cerebellum of TG mice. The color-gradient indicates the adjusted *p*-value for the enrichment. Numbers in parentheses indicate the number of identified proteins in each category. Dot size corresponds to “protein count for each group”/“total protein count for each category.” **(B)** Heatmap showing the expression across tissues (TPM, transcript per million) of the cerebellar proteins found to be differentially abundant in our study, showing their cerebellar enrichment. **(C)** Enrichment analysis on the cerebellar-enriched proteins using the GO annotations.

We used label-free quantitative mass spectrometry-based proteomics (LC-MS/MS) to characterize the cerebellum proteome on whole protein extracts from TgDyrk1A (TG) and wild-type (WT) mice, treated or not with EGCG-containing green tea extract. In total, we quantified 3,608 proteins whose abundances were fitted into a linear model to evaluate changes related to the genotype, the treatment, and their interaction. We set a threshold of adjusted *p*-value (Benjamini-Hochberg correction) of 0.05 and a minimum absolute log2 fold change of 0.3 to consider a protein to be significantly changing (Table 1; Supplementary Figure S1; Vizcaíno et al., 2014). Out of the 3,608 quantified proteins, in the cerebellum of TG mice we found 90 significantly upregulated and 99 downregulated proteins (adjusted *p*-value <0.05; Table 1) with respect to WT. We also found proteins that were preferentially detected in one condition: 31 proteins were present exclusively in TG samples, and 53 proteins were exclusively detected in WT samples, thus making a total of 273 proteins with differential abundance. Among the proteins with altered abundance, 16 of them are known to be direct DYRK1A interactors or targets (Table 1). Gene ontology analysis of the deregulated proteins in the cerebellum of TG mice revealed a significant enrichment for “cytochrome complex,” and in general in the oxidative phosphorylation system (OXPHOS; Figure 1A). We also observed that 16 of the differentially abundant proteins had previously been reported to be preferentially expressed in the cerebellum (Figure 1B; Jain and Tuteja, 2019). These proteins were enriched in categories that included myelination and axon ensheathment (Figure 1C).

**Table 1:** Differentially abundant proteins in TG Dyrk1A mouse cerebellum.

	Detected proteins (*n*)	Upregulated (*n*)	Present in TG, absent in WT	Downregulated (*n*)	Absent in TG, present in WT	DYRK1A targets	DYRK1A targets (in first interactors)
TG.NT-WT.NT	3,608	90	31	99	53	Phb2, Eef1b2, Creb1, Plbd2, Wdr47, Sfpq, Eif2b1, Prkar1a, Eef1b	Fhl2, Prkaca, Traf2, Stat3, Psen1, Ywhag, Kpnb1, Smarca4, Myo1c
WT.EGCG-WT.NT	3,554	142	22	60	32	Prkaca, Tra2b, Prkacb, Creb1, Tanc1, Wdr26, Phyhip, Eif2b1, Srsf10, Sf3b1	Trp53, Egr2, Braf, Traf2, Stat3, Gli1, Ywhag, Notch1, Ywhab, Prkar1a, Gsk3b, Tp53
TG.EGCG-TG.NT	3,603	97	47	110	35	Anapc1, Tanc1, Plbd2, Srsf2, Srsf1, Tomm34, Srsf10	Egr2, Polr2a, App, Map1b, Grin2a, Traf2, Stat3, Gli1, Psen1, Mapk1, Notch1, Smarca4, Ablim1, Wasl, Ywhab, Myo1c, Ap3b1
(TG.EGCG-TG.NT)-(WT.EGCG-WT.NT)	3,443	122	23	70	11	Phb2, Ywhag, Srsf2, Srsf1, Eif2b1, Prkar1a	Fhl2, Prkaca, Egr2, Polr2a, Traf2, Stat3, Psen1, Kpnb1, Smarca4, Grb2, Rbl2, Wasl, Ywhab, Myo1c
Rescued by EGCG	123	NA	NA	NA	NA	Phb2, Eef1b2, Plbd2, Eef1b	Stat3, Psen1, Kpnb1, Myo1c

**Figure 2:**
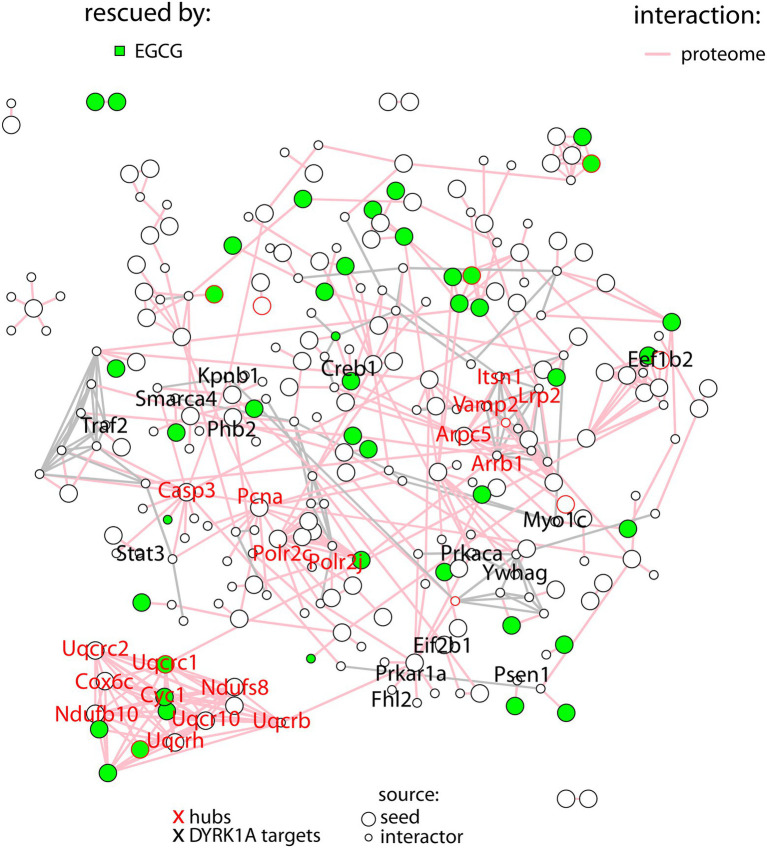
Protein–protein interaction network with the proteins differentially abundant in TG mice (*seed* proteins), represented as big nodes and their primary interactors (small nodes). Interactions between two *seed* proteins are depicted as pink edges. Interactions between interactors are shown in gray. Proteins rescued upon treatment are colored in green. The names of the hubs are depicted in red, the names of DYRK1A targets in black.

In order to better understand the molecular pathways affected by DYRK1A overexpression in the cerebellum, we built a protein–protein interaction network with the proteins deregulated in TG cerebellum. A total of 139 proteins (out of the 273) were involved in known protein–protein interactions (PPI, STRING database score > 0.4), and those were established as *seed* proteins (Figure 2). Then, we added their 130 *bona fide* primary interactors previously reported in the STRING database, creating a final network of 269 interacting proteins and a total of 511 interactions.

The number of interactions per protein (node degree) in the network followed a log-normal right-skewed distribution (Supplementary Figures S1A,B). Using this distribution, we detected 18 “protein hubs,” defined as those appearing in the 10% right tail of the node-degree distribution (Supplementary Figures S2A,B) with an average node degree of 12 (from 11 to 14). Of these 18 hub proteins, 12 were *seed* proteins of the TG network, while the others were primary interactors (Table 2). Moreover 5 of these hubs, Ndufs8 (NADH:Ubiquinone Oxidoreductase Core Subunit S8), Ndufb10 (NADH:Ubiquinone Oxidoreductase Subunit B10), Uqcrc2 (ubiquinol-cytochrome c reductase complex (complex III) of the mitochondrial respiratory chain), Cyc1 (OXPHOS complex III) and Cox6c (OXPHOS complex IV), formed a cluster with most of their interactors in common. Interestingly, the hubs and ~10% of their common interactors were rescued by EGCG-containing green tea extract (Supplementary Figures S2C,D).

**Table 2:** Protein hubs in TG Dyrk1A cerebellum network.

Interactions (*n*)	Value of *p*	Symbol	Uniprot ID	Description	Rescued	Seed
14	0.05806	Casp3	P70677	Caspase 3		Yes
13	0.06702	Vamp2	P63044	Vesicle-associated membrane protein 2		
13	0.06702	Uqcrb	Q9D855	Ubiquinol-cytochrome c reductase binding protein		
13	0.06702	Arpc5	Q9CPW4	Actin related protein 2/3 complex, subunit 5		Yes
13	0.06702	Uqcrh	P99028	Ubiquinol-cytochrome c reductase hinge protein	Yes	Yes
13	0.06702	Polr2c	P97760	Polymerase (RNA) II (DNA directed) polypeptide C		Yes
12	0.07788	Uqcrc1	Q9CZ13	Ubiquinol-cytochrome c reductase core protein 1	Yes	Yes
12	0.07788	Pcna	P17918	Proliferating cell nuclear antigen	Yes	Yes
12	0.07788	Polr2j	O08740	Polymerase (RNA) II (DNA directed) polypeptide J		
12	0.07788	Uqcr10	Q8R1I1	Ubiquinol-cytochrome c reductase, complex III subunit X		Yes
12	0.07788	Arrb1	Q8BWG8	Arrestin, beta 1		
12	0.07788	Itsn1	Q9Z0R4	Intersectin 1 (SH3 domain protein 1A)		
11	0.0912	Cox6c	Q9CPQ1	Cytochrome c oxidase subunit 6C	Yes	Yes
11	0.0912	Cyc1	Q9D0M3	Cytochrome c-1	Yes	Yes
11	0.0912	Uqcrc2	Q9DB77	Ubiquinol cytochrome c reductase core protein 2	Yes	Yes
11	0.0912	Ndufb10	Q9DCS9	NADH:ubiquinone oxidoreductase subunit B10	Yes	Yes
11	0.0912	Ndufs8	Q8K3J1	NADH:ubiquinone oxidoreductase core subunit S8	Yes	Yes
11	0.0912	Lrp2	A2ARV4	Low density lipoprotein receptor-related protein 2		
10	0.10771	Cox5a	P12787	Cytochrome c oxidase subunit 5A	Yes	Yes
10	0.10771	M6pr	P24668	Mannose-6-phosphate receptor, cation dependent		Yes
10	0.10771	Ndufa1	O35683	NADH:ubiquinone oxidoreductase subunit A1	Yes	Yes
10	0.10771	Csnk1d	Q9DC28	Casein kinase 1, delta		Yes
10	0.10771	Plk1	Q07832	Polo like kinase 1		
10	0.10771	Ikbkb	O88351	Inhibitor of kappaB kinase beta		
10	0.10771	Prkar1a	Q9DBC7	Protein kinase, cAMP dependent regulatory, type I, alpha		Yes
10	0.10771	Cttn	Q60598	Cortactin		
9	0.12845	Birc3	O08863	Baculoviral IAP repeat-containing 3		
9	0.12845	Crebbp	P45481	CREB binding protein		
9	0.12845	Cox4i1	P19783	Cytochrome c oxidase subunit 4I1	Yes	Yes
9	0.12845	Pacsin1	Q61644	Protein kinase C and casein kinase substrate in neurons 1		
9	0.12845	Pacsin3	Q99JB8	Protein kinase C and casein kinase substrate in neurons 3		Yes
9	0.12845	Xiap	Q60989	X-linked inhibitor of apoptosis		
9	0.12845	Pacsin2	Q9WVE8	Protein kinase C and casein kinase substrate in neurons 2		
9	0.12845	Birc2	Q62210	Baculoviral IAP repeat-containing 2		
9	0.12845	Creb1	Q01147	camp responsive element binding protein 1		Yes
8	0.1549	Tnfrsf1a	P25118	Tumor necrosis factor receptor superfamily, member 1a		
8	0.1549	Rpl28	P41105	Ribosomal protein L28		Yes
7	0.18922	Ranbp2	Q9ERU9	RAN binding protein 2		
7	0.18922	Prkaca	P05132	Protein kinase, cAMP dependent, catalytic, alpha		
7	0.18922	Mylk	Q6PDN3	Myosin, light polypeptide kinase		
7	0.18922	Rhoq	Q8R527	Ras homolog family member Q		
7	0.18922	Chuk	Q60680	Conserved helix-loop-helix ubiquitous kinase		
7	0.18922	Baiap2	Q8BKX1	Brain-specific angiogenesis inhibitor 1-associated protein 2	Yes	Yes
7	0.18922	Ndufs5	Q99LY9	NADH:ubiquinone oxidoreductase core subunit S5	Yes	Yes

### 3.2. Green tea extracts partially rescue the molecular processes disrupted by DYRK1A overexpression

**Figure 3:**
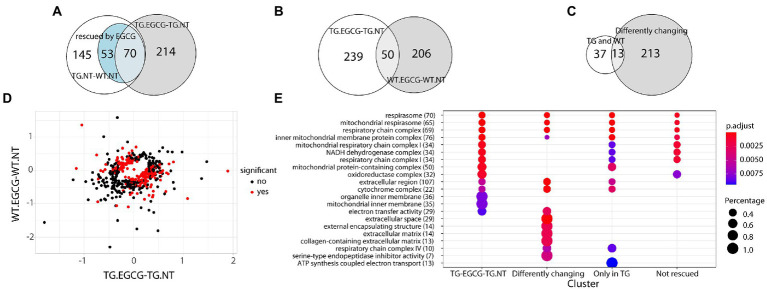
Dyrk1A overexpression extend beyond the network and differently affect wt and TgDYRK1A mice **(A)** Venn diagram showing the overlap between proteins changing their abundance in TG mice, protein changing upon EGCG-containing green tea extract treatment in TG mice, and the ones rescued. **(B)** Venn diagram showing the overlap between the proteins changing in TG mice upon treatment and the ones changing in WT. **(C)** Venn diagram showing the overlap between protein changing in both TG and WT mice upon EGCG-containing green tea extract treatment and the ones exhibiting a genotype-specific response to EGCG-containing green tea extract treatment **(D)** Plot comparing protein fold changes upon EGCG treatment in TG mice (*x*-axis), and wild-type mice (*y*-axis). Proteins with a significant interaction in the contrast *(TG.EGCG-TG.NT)* − *(WT.EGCG-WT.NT)*, where NT stands for “not treated,” are indicated as red dots. **(E)** Enrichment analysis for biological processes, molecular functions, and cell components upon EGCG-containing green tea extract treatment in TG. The color-gradient indicates the adjusted *p*-value for the enrichment. Numbers in parentheses indicate the number of identified proteins in each category. Dot size corresponds to (protein count for each group)/(total protein count for each category). Only categories with an FDR < 1% are shown.

**Figure 4:**
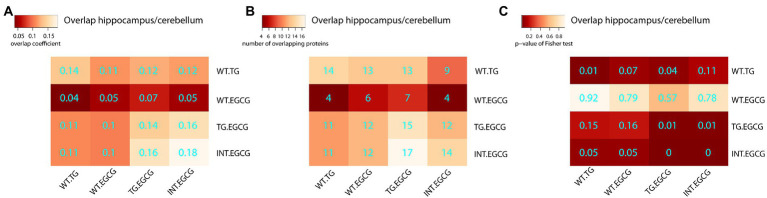
Overlaps of differentially abundant proteins across experimental conditions. Heatmap showing the overlap between differentially abundant proteins in the cerebellum and hippocampus. **(A)** The Szymkiewicz–Simpson overlap coefficient is depicted in cyan; **(B)** the number of overlapping proteins; **(C)** the *p*-values of the exact Fisher test. TG, TG mice. WT, wild-type mice. NT, not treated, EGCG treated with green tea extracts.

Next, we treated TG mice with green tea extract, which has inhibitory properties on DYRK1A kinase activity, and compared its cerebellar proteome to that of TG not-treated mice. We first analyzed rescued proteins, e.g., those with compromised abundances in TG mice that were restored to WT levels upon the treatment, and found that 50% of the original abundance level of 123 proteins (45% of those differentially abundant) were restored. For 70 of these proteins, the effect of the treatment was significant (TG_EGCG vs. TG_NT; Figure 3A). Rescued proteins were enriched in categories connected with mitochondria and oxidative phosphorylation. However, the treatment had a much wider effect on the proteome in TG mice, as it significantly changed the abundance of a total of 289 proteins. These proteins were also mainly involved in mitochondrial pathways connected to oxidative phosphorylation and the respirasome. Noteworthy, green-tea extract also affected 256 proteins in WT mice, of which only 50 were in common with TG-treated mice (Figure 3B). However, enrichment analysis of the proteins changing in WT upon green tea-extract treatment showed no enrichment in any significant categories, thus suggesting EGCG action was more meaningful in TG mice, as was also observed in previous work in the hippocampus (De Toma et al., 2019). We found 226 proteins with significant changes in abundance upon treatment in both genotypes (Figure 3C). However, those showed a divergent response to green-tea treatment, with protein abundance increased in one genotype and reduced in the other upon green-tea treatment (red dots in Figure 3D). These 226 proteins were enriched in the mitochondrial respirasome but also in “extracellular space” and in “serine-type endopeptidase inhibitor activity” (Figure 3E). Taking advantage of our previously published proteomic dataset of the hippocampus of the same type of mice, we could also compare the proteomic changes due to DYRK1A overexpression in both regions. In the hippocampus, changes were mainly related to antioxidant activity categories and involved a lower number of proteins in the hippocampus (98/2685 for the hippocampus (De Toma et al., 2019), versus 273/3608 in the cerebellum). Indeed, we detected significant overlaps of 14%–18% corresponding to 14 proteins differentially abundant both in the cerebellum and the hippocampus when looking at the genotype contrast and 15 in the treatment contrast in TG (Figure 4). However, when looking at the treatment contrast in WT, the overlap was not significant (5%, corresponding to 6 proteins), indicating that the proteins changing in WT upon EGCG-containing green tea extract treatment are less consistent, and thus, probably randomly deregulated.

### 3.3. Mitochondrial DNA copy number per cell in the Declive neuron-rich area of the cerebellum is increased in TG mice

**Figure 5:**
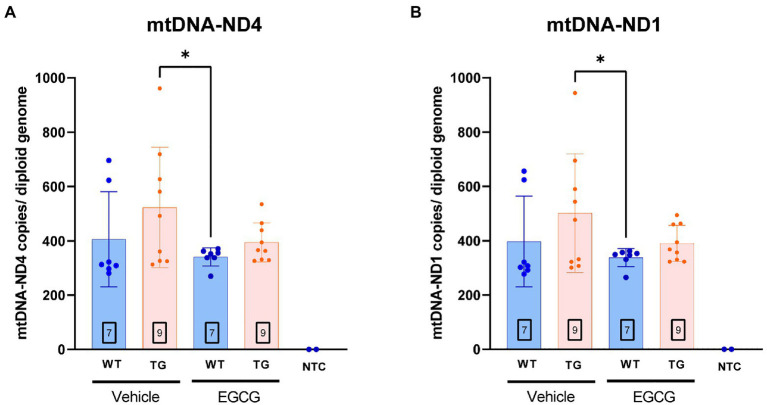
mtDNA copy number per cell is increased in TG mice. mtDNA copy number per cell in the Declive neuron-rich area of the cerebellum. Quantification of mitochondrial DNA copy number per diploid genome was performed with a multiplex digital PCR with different primer pairs targeting opposed regions of the mtDNA sequence: **(A)** the ND4 coding region and **(B)** the ND1 region. The n number of animals is within squares. Results are expressed as mean ± SD. *p < 0.05.

In order to validate whether DYRK1A overexpression leads to mitochondrial dysfunction in TG mice, we measured the number of mitochondrial DNA (mtDNA) copy number and nuclear genomes in the different experimental groups. mtDNA copy number of ND1 (*p* = 0.16) and ND4 (*p* = 0.13) showed a tendency to be higher in TG mice compared to WT mice. mtDNA copies of ND1 (*p* = 0.03) and ND4 (*p* = 0.02) were significantly higher in TG mice compared to WT mice treated with EGCG (Figures 5A,B). TG mice treated with EGCG-containing green tea extract showed reduced, albeit non-significant, mtDNA copy number of ND1 (*p* = 0.11) and ND4 (*p* = 0.07) compared to non-treated TG mice.

## 4. Discussion

*DYRK1A* is one of the most important candidate genes to explain Down syndrome (DS) neuropathology. DYRK1A immunostaining is found in the cerebellum (Martí et al., 2003), suggesting a possible involvement of this kinase in the cerebellar physiology and pathology, which might be involved in DS motor, and learning phenotypes. However, the proteomic modifications due to *Dyrk1A* overexpression in the cerebellum had not been yet explored. Using mass-spectrometry-based proteomic we detected important proteomic alterations in Tg*Dyrk1A* (TG) cerebellum with over 200 proteins showing changes in abundance, a much higher number than what was detected in the hippocampus (De Toma et al., 2019). The most significant changes indicate mitochondrial dysfunction as a crucial factor in the pathogenesis of cerebellar impairment in TG mice. Interestingly, treatment with green tea extracts rescued the level of numerous proteins, especially those involved in mitochondrial function.

*Dyrk1A* overexpression in the cerebellum of TG mice altered mitochondrial pathways with a significant enrichment for “cytochrome complex,” and in general in the oxidative phosphorylation system (OXPHOS). In fact, oxidative stress and mitochondrial dysfunction are involved in DS and have been reported in *in vitro* and *in vivo* models of DS. The OXPHOS has five mitochondrial respiratory chain complexes (complexes I-V), complex I (NADH–ubiquinone oxidoreductase), complex II (succinate dehydrogenase), complex III (ubiquinone-cytochrome c oxidoreductase), complex IV (cytochrome C oxidase), and the ATP synthase. We detected a differential abundance of ubiquinol:cytochrome c oxidoreductase (complex III), ATP synthase (complex V), and NADH:ubiquinone oxidoreductase (complex I). In previous proteomic studies, complex III core protein 1 was significantly reduced in the temporal cortex of AD patients while complex V beta chain and protein levels of complex I 30-kDa subunit were significantly reduced in the cerebral cortex of adult (Kim et al., 2000), and fetal DS brain (Kim et al., 2000, 2001). Our results support previous findings from Valenti et al. who found that deficits in the OXPHOS system in DS fibroblasts and lymphoblastoid cells lead to energy deficit and a dramatic increased ROS production in mitochondria (Valenti et al., 2010, 2011), and the severe deficits in mitochondrial bioenergetics in hippocampal neural progenitor cells (NPCs) of Ts65Dn mice (Valenti et al., 2016). These disturbances of enzymes of the mitochondrial electron transport chain may lead to a reduction in mitochondrial energy production, altered mitochondrial morphology, and increased fragmentation (Piccoli et al., 2013) that could be linked to cerebellar hypoplasia (Guidi et al., 2011) in DS. In fact, deregulated mitochondrial oxidative phosphorylation complexes are an early marker of cognitive decline in DS and AD (Alldred et al., 2021). Here, we tested if gene deregulation caused by the sole overexpression of *Dyrk1A* would influence mtDNA replication (Clayton, 1992). mtDNA is a double-stranded DNA molecule encoding for 13 structural peptide subunits of the oxidative phosphorylation system along with 24 RNA molecules required for mitochondrial protein synthesis (Anderson et al., 1981; Andrews et al., 1999). It has been described that mutations in mtDNA can cause defects in structural proteins, impair mitochondrial RNA synthesis, and cause deficiencies in the respiratory chain (Sommerville et al., 2014; Lightowlers et al., 2015). Other DS mouse models, such as Ts1Cje, show decreased mitochondrial membrane potential and ATP production, and increase reactive oxygen species, although few studies have explored the cerebellum. Moreover, irregular shaped mitochondria have been reported in cerebellar neurons from Ts16 mice (Bersu et al., 1998).

We found a trend to higher mtDNA copy number of ND1 and ND4 in TG mice compared to WT mice. Our findings suggest that *Dyrk1A* overexpression would increase mtDNA copy number contributing to mitochondrial dysfunction leading to the alterations previously reported at the cerebellar level (Altafaj et al., 2008) in TG mice. Supporting this possibility, it was recently reported that DYRK1A is able to activate mitochondrial import machinery (Walter et al., 2021).

Remarkably, treatment with EGCG-containing green tea extract partially recovered the mtDNA copy number in TG mice. The stabilizing effect of EGCG on mtDNA copy number is consistent with previous findings showing that EGCG restored mtDNA copy number in hippocampal neuronal precursor cells (NPCs) from Ts65Dn mice (Valenti et al., 2016). In these cells, EGCG increased the number of mtDNA copies lowered by Ts65Dn to control levels (Valenti et al.). In contrast, the results reported here show that, in the cerebellum, EGCG lowers the number of mtDNA copies increased by Dyrk1A back to control levels. This difference is most likely due to brain region-specific regulation of mtDNA copy number. The amount of mtDNA copies per cell differs in almost one order of magnitude between the hippocampus and cerebellar cells (mtDNA) copy number relative to whole brain median = 0.7 vs. 0.09 (Fuke et al., 2011). Thus, Dyrk1A overexpression or Ts65Dn may alter mtDNA copy numbers differently in each brain region. Altered mtDNA copy number has been associated with different neurodegenerative disorders (Wei et al., 2017), and normalizing the number of mtDNA copies in different neurodegenerative diseases has been hypothesized to have a beneficial effect (Filograna et al., 2021). However, the mechanisms of brain region and cell-type specific regulation of mtDNA copy number are still unknown and further studies are necessary to determine the molecular mechanisms of selective vulnerability of neurodegeneration. Nonetheless, the present results suggest that Dyrk1a overexpression alters mtDNA copy number in the cerebellum and provide evidence to suggest that EGCG prevents alteration of mtDNA copy number set point in this brain region.

Treatment with EGCG-containing green tea extract also rescued the levels of 123 proteins (45%) to WT levels in TG mice. Rescued proteins were enriched in categories connected with mitochondria and oxidative phosphorylation. However, the effects of the treatment were mainly genotype-specific, as many proteins showed an opposite direction of change upon green-tea treatment in WT compared to TS (note the red dots in Figure 3D). These proteins were enriched in the mitochondrial respirasome but also in “extracellular space” and in “serine-type endopeptidase inhibitor activity” (see Figure 3E). Interestingly, comparing our previous proteomic study in the hippocampus, we observed that *Dyrk1A*-overexpression leads to region-specific proteomic dysregulation in the cerebellum.

In summary, our proteomic study revealed that *Dyrk1A* overexpression affects protein levels related with antioxidant activity and cerebellar proteins involved in oxidative phosphorylation system and cytochrome complex, suggesting a mitochondrial dysfunction. This was further emphasized by the increase of mtDNA copy number in TG cerebellum, which may explain mitochondrial dysfunction. EGCG-containing green tea extract treatment, which was already effective in rescuing the proteome alterations in the hippocampus, was also able to partially restore cerebellar protein levels and normalized the increased mtDNA levels that were found in non-treated TG mice.

## Data availability statement

The datasets presented in this study can be found in online repositories. The names of the repository/repositories and accession number(s) can be found at: http://www.proteomexchange.org/, PXD030772 and directly at https://github.com/Ilarius/TgDyrk1A_protemics_cerebellum.

## Ethics statement

The animal study was reviewed and approved by Comité Ético de Experimentación Animal del PRBB (CEEA-PRBB), Parc de Recerca Biomédica de Barcelona.

## Author contributions

MO performed the proteomics experiment and made some of the analyses. IT performed the analyses, wrote the manuscript, and prepared all the figures. ÁF-B prepared the figures and did the validation experiments together with AC, LB, and RT. MD and ES conceived the project, supervised the experiments and analyses, and revised the manuscript. All authors contributed to the article and approved the submitted version.

## Funding

This research was funded by the Agencia Estatal de Investigación (PID2019-110755RB-I00/AEI/10.13039/501100011033), the European Union’s Horizon 2020 Framework Programme under grant agreement no 848077. This reflects only the author’s view and the European Commission is not responsible for any use that may be made of the information it contains. Jerôme Lejeune Foundation (grant number 2002), NIH Blueprint for Neuroscience Research (grant number: 1R01EB 028159-01), Marató TV3 (#2016/20-30), and EU Joint Programme—Neurodegenerative Disease Research (Heroes AC170006). The CRG/UPF Proteomics Unit is part of the Spanish Infrastructure for Omics Technologies (ICTS OmicsTech), and it is supported by “Secretaria d’Universitats i Recerca del Departament d’Economia i Coneixement de la Generalitat de Catalunya” (2017SGR595). The CRG acknowledges the support of the Spanish Ministry of Science and Innovation to the EMBL partnership, the Centro de Excelencia Severo Ochoa, and the CERCA Programme/Generalitat de Catalunya. The CIBER of Rare Diseases (CIBERER) is an initiative of the ISCIII.

## Conflict of interest

The authors declare that the research was conducted in the absence of any commercial or financial relationships that could be construed as a potential conflict of interest.

## Publisher’s note

All claims expressed in this article are solely those of the authors and do not necessarily represent those of their affiliated organizations, or those of the publisher, the editors and the reviewers. Any product that may be evaluated in this article, or claim that may be made by its manufacturer, is not guaranteed or endorsed by the publisher.

## Supplementary material

The Supplementary material for this article can be found online at: https://www.frontiersin.org/articles/10.3389/fnmol.2022.1015220/full#supplementary-material
