# Supplementary material for: Proteomic profiling reveals mitochondrial dysfunction in the cerebellum of transgenic mice overexpressing DYRK1A, a Down syndrome candidate gene
Source: Front Mol Neurosci. 2022 Dec 15;15:1015220. doi: 10.3389/fnmol.2022.1015220 (PMC9800213; doi:10.3389/fnmol.2022.1015220)
Supplement: Supplementary file 1 [file Data_Sheet_1.docx]

# Supplementary Material

**
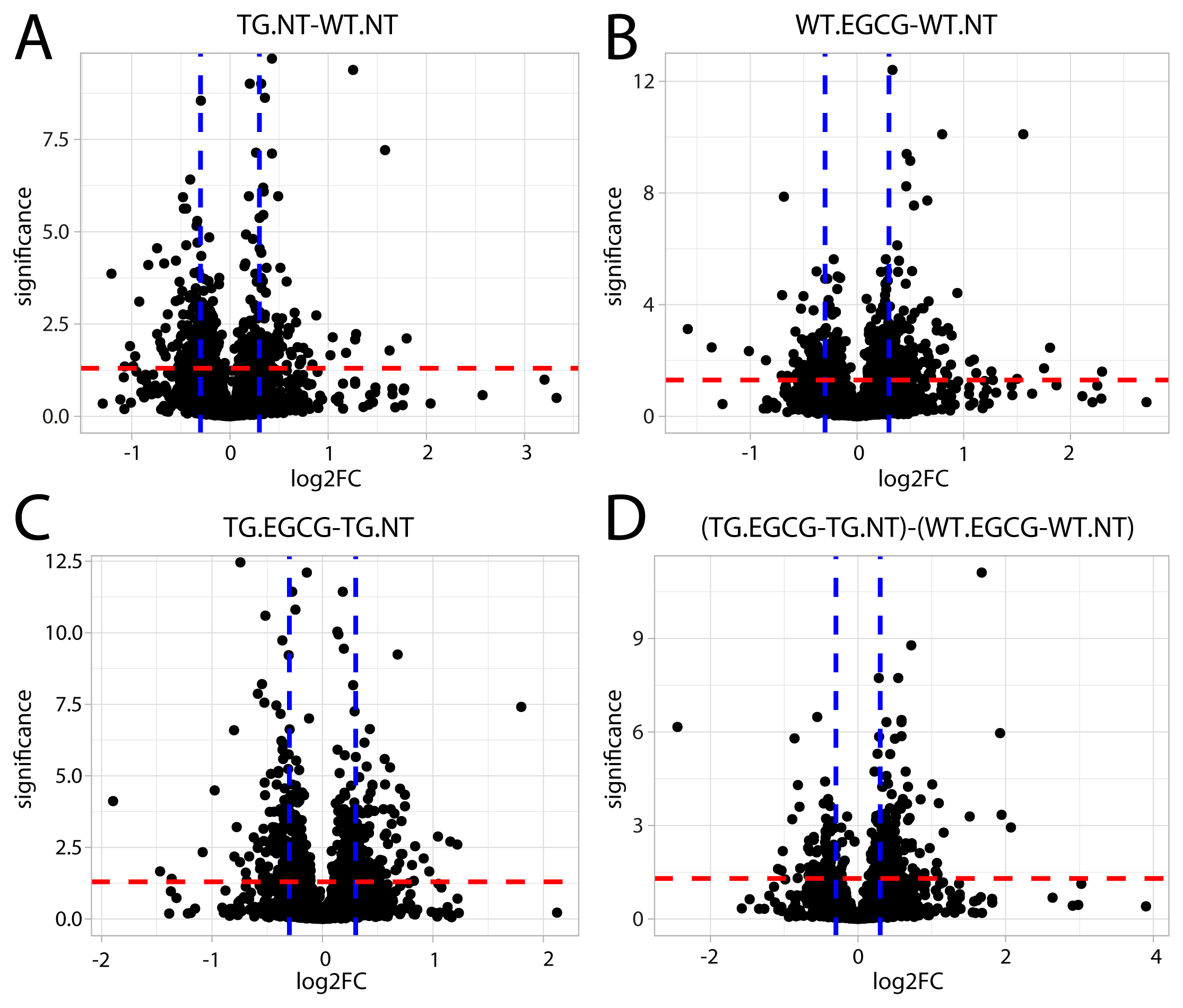
Supplementary Figure 1.** Volcano plots showing the log2 Fold change on the x axis and the significance (-log10(adjusted-p value)) on the y-axis in the four different contrasts examined: (**A)** TG.NT-WT-NT; (**B)** WT-EGCG-WT.NT, (**C)** TG.EGCG-TG.NT, and (**D)** (TG.EGCG-TG.NT)-(WT.EGCG-WT.NT).

**
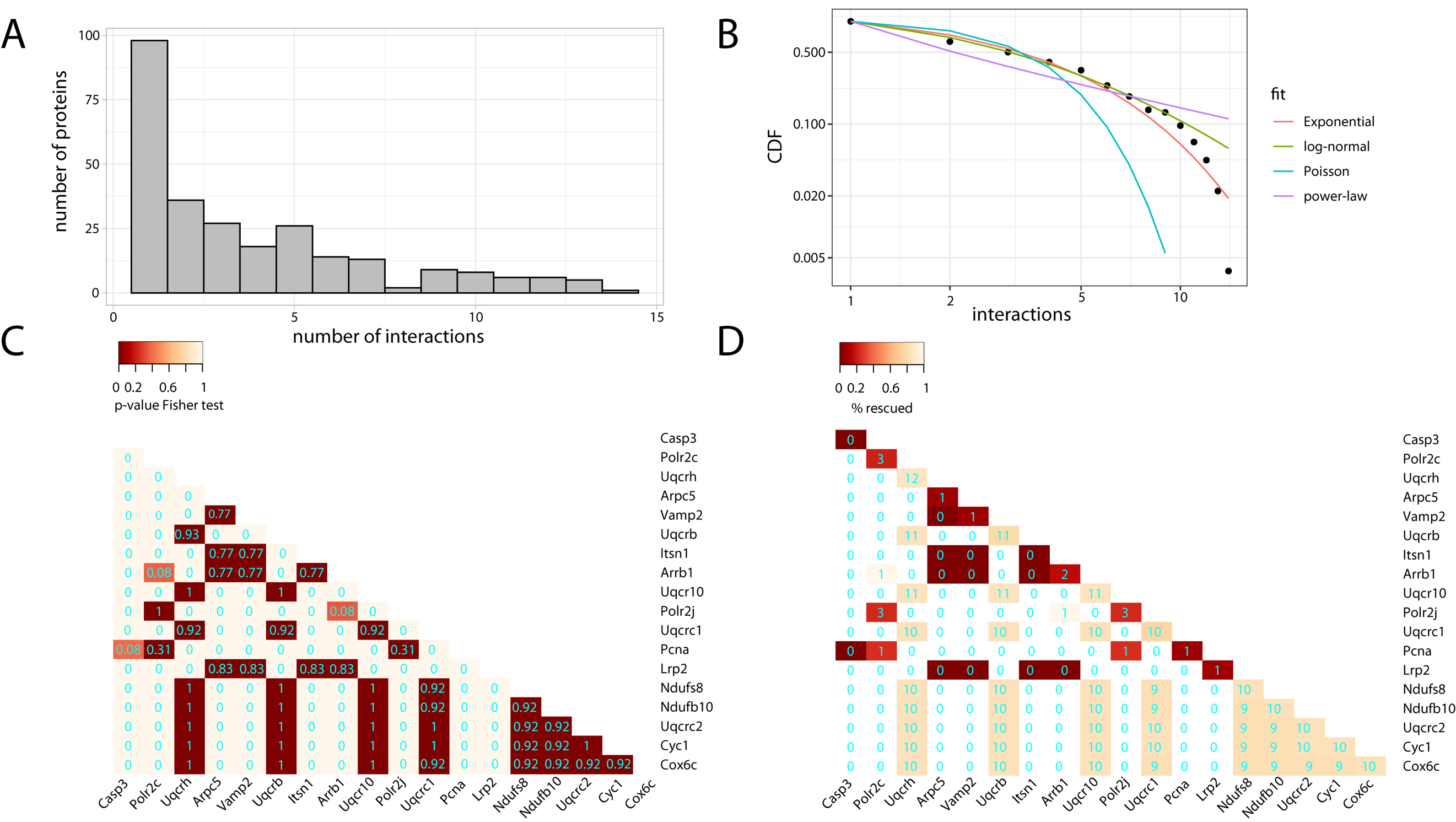
Supplementary Figure 2. (A)** Histogram of the number of interactions per protein in the network. (**B)** Cumulative distribution functions (y-axis) versus the number of observed interactions). (**C)** Heatmap showing the overlap of the hub’s interactions (left) and the percentage of commonly rescued proteins in these overlaps (right).
